# Mechanical Treatment of Cleft Palate, Obturators, and Artificial Velum

**Published:** 1883-12

**Authors:** Oscar Booth

**Affiliations:** Creston, Iowa.


ARTICLE V.

### MECHANICAL TREATMENT OF CLEFT PALATE, OBTURATORS, AND ARTIFICIAL VELUM.

BY OSCAR BOOTH, D. D. S., CRESTON, IOWA.

(Read before Iowa State Dental Society, May, 1883.)

Among the ancients a mistaken notion prevailed that the sense of taste resided in the roof of the mouth; and so, according to their custom of naming all objects from some peculiar characteristic which they possessed, they called the oral arch the "palate," a word signifying to perceive by the taste. Custom often becomes law, and so it happens that the use of the word is retained. The hard, bony structure of the roof of the mouth is essentially an arch. Its continuation posteriorily into a soft curtain-like structure is also essentially a valve. To speak of the first as the oral arch, and of the second as the naso-pharyngeal valve, would at once convey a clear idea of the parts; whereas the word "palate" signifies nothing, except to convey a false impression. Indeed the word "palate," among the common people, means nothing more or less than the pendant portion of the soft palate, named the Uvula. Now with these two parts, the oral arch and the naso-pharyngeal valve, we have to do in discussing the subject of obturators and mechanical treatment of cleft palate. We submit to the word "palate" because we must, since custom becomes law; but we enter our protest against the use of the word as meaningless, signifying neither shape, location, nor characteristic.

In the vicissitudes of human life it sometimes happens that one is born with a defect in the hard or soft palate. This class of defects we call congenital. They are due to a suspended or mal-development of the part during the early period of intra-uterine life.

Another class of defects of the palatine arch arise from some disease, and these we term accidental lesions of the palate. They are easily distinguished from the congenital by their irregularity, whereas congenital cleft is always found to follow the median line, and present smooth, round edges. Congenital cleft may be a partial or entire bifurcation of the soft palate, or it may extend through both the soft and hard palate from the extremity of the uvula to the junction of the median line with the intermaxillary suture, where it either stops or is deflected to one side or the other, causing fissure of the lip. Accidental or acquired lesion of the palate is usually of syphilitic origin, and is slight or extensive according to the scope of the disease which has caused it. The effects of disease may have been limited to a small orifice in the soft palate which in time, with proper treatment, may be closed by nature. On the other hand unchecked syphilis knows no bounds, but, like the destroying angel, strikes down everything in its pathway, causing injuries which the most consummate skill may not succeed in relieving. Illustrations of the terrible effects of this disease on the palatine organs may be found in Kingsley's "Oral Deformities," and, indeed, in almost any work on oral surgery. According to Kingsley the first recorded definite suggestion of a piece of mechanism to act as a palatine obturator is that of Alexander Petronius, who preceded the celebrated Ambrose Pare by a few years. The first definite description of an obturator was by Ambrose Pare, whose first work was published in 1541. We see that obturators are no new invention. Crude at first, they consisted of cotton, wax, or any pliable material that would answer for a plug. Then came the little instrument representing a button stud, with a piece of sponge fastened to the end to be passed through the aperature, which, when moistened, as Pare says, "with the moisture distilling from the brain, will become more swollen and puffed up, so that it will fill the concavity of the palate, that the artificial palate cannot fall down, but stand fast and firm as if it stood by itself. According to Kingsley this little device, with a slight change in the manner of attaching the sponge by Laurence Heister, in 1756, was the only method used during a period of two hundred years. Then in 1756, through Bourdett came the idea of using sheet metal to arch over the vault of the palate supported and held in place by silk ligatures about the teeth. Gold clasps were substituted for these silk ligatures by M. Delabarre in 1820.

No improvement on the use of sheet metal for bridging across a break or gap in the hard palate caused by disease has ever been made. Experience has taught that any substance which fills up the aperature and is allowed to press upon its surrounding edges will be likely to cause their absorption and consequent enlargement of the opening, to say nothing of the likelihood of still more serious trouble arising from inflammation. But provided the break be traumatic, as a gun-shot wound, and is large so as never to be closed by nature, the crasis of the blood being good, and all the parts in a healthy condition, then advantage may be taken of one or more openings through the hard palate, or through the alveola border into the antrum, for giving stability to the plate by forming hollow nubs of metal or vulcanized rubber upon its upper surface to fill these spaces. A variety of lesions of this character may be seen illustrated in Kingsley's " Oral Deformities," Garretson's " Oral Surgery," and various other works of like character.

No two accidental lesions of the hard palate are alike, yet the principle of covering them over and restoring the form of the arch with a metal plate like gold or silver, is correct. With these few remarks we leave the subject of accidental lesions of the palate because they have been treated upon in the fullest manner by those who wield abler pens than ours, and pass at once to the consideration of those defects of the palatine arch which are far more difficult of correction, namely:—those of congenital origin, difficult for the same reason that it is difficult to learn to write. No man can write without a pen, no man can talk without a palate. No man can write with a pen unless he first learns to use it. Education of the muscles of the fingers and arm are necessary before the crudest forms of writing can be produced. Close application and persevering practice alone can make the skilled penman, so in like manner is it with a palate, be it natural or artificial, if one would become proficient in uttering intelligent language. Another proposition ;—A poor pen in the hands of a skilled penman may be made to produce words that can be read and understood; so also a poorly constructed artificial palate with intelligence and skill in its use, may enable its possessor to utter words that can be understood, but to those who suffer from congenital cleft of the palate, clear distinct utterance, and those agreeable tones of voice which so delight the ear are only possible, first to him who has a skillfully constructed instrument to supply the defect, and second to his own patient, persevering, well directed efforts in learning to use it. How shall this be accomplished? The answer is simple and yet practically of such difficulty that the greatest nicety of skill will scarcely succeed on first trial. Take for example a typical case of congenital cleft. The bifurcation extending from the extremity of the uvula to the intermaxillary suture, the borders of the gap widely separated. Primarily two objects are desirable, first to give the faculty of perfect deglutition, and second to give the patient the abilily to learn to articulate sounds correctly. Other reasons exist for the construction of an artificial palate which more nearly concern the patient's life. The patient comes to us for relief, what shall be done? Let us proceed now step by step, if we would succeed and avoid confusion and annoyance. The first thing to be thought of, is to bridge across the gap in the hard palate with a gold or silver plate, so that the form of the vault of the arch shall be given a symetrical contour corresponding with what the normal condition would have been had the cleft never existed. To do this we take an impression of the cleft, extendiug as far back as the posterior border of the hard palate, allowing the plaster to run over the floor of the nares Now with a thin curved spatula, trim its under surface from the border of the lowest aside of the gap to the opposite side of the vault in such a manner that there shall be regular contour to the arch. The impression may be most easily removed by pushing back toward the pharynx. Its upper surface should be trimmed, so that only a thin edge on one side will over-hang the floor of the nares. It is desirable that this model should be reproduced in vulcanized rubber, to avoid continual annoyance from breaking its edges in the process of making dies and counter-dies, besides rubber may be polished perfectly smooth, and so be much better for sand moulding. This plate must necessarily be swaged in two or more parts, and these parts united with solder. When this is done the piece may be placed in position, and an impression taken precisely as for a whole or partial set of teeth, and a plate swaged accordingly, to which can be attached any teeth that may be required, or a clasp or clasps to sustain the plate in position. The two parts now placed in the mouth, will be brought into their proper relation, when by putting plaster on the posterior border, they may be removed and soldered, thus completing the first part of the plate.

The second step is to make a velum which should also be made of thin sheet metal. To this end we first take a piece of wax and work it into a shape somewhat resembling a chestnut, letting it extend from the posterior border of the obturator, already in position to within three-sixteenths of an inch of the superior constrictor of the pharynx, bevelling its sides from below upward and outward, so as to overlie the Levator palati on either side. The under surface should be arched somewhat in the form of the natural velum at rest, and the superior surface made oval approximating the natural velum in an elevated position. By successive efforts at trimming and introducing the model to its place, it may be brought to just the right proportions so as not to press unduly upon the surrounding tissues, but at the same time be acted upon by the palate and superior constrictor muscles, during the act of deglutition, or articulating sounds, thus forming a complete valve between the posterior nares and the pharynx. Having obtained a perfect model of the velum, we proceed with the work of making dies and counter-dies for swaging the plate in two halves, so that when united with solder it will form a hollow bulb, at once light, strong, and durable, far superior to vulcanized rubber in giving tone to the voice. All that remains now to be done is to attach the velum to the obturator with a hinge. While the obturator is in position in the mouth, carry the velum back of it and hold it in place with an instrument in the left hand, now with a sharp excavator, scratch both the obturator and velum so as to indicate their relative positions when removed from the mouth. Invest in the plaster and solder the hinge to the parts, using gold joint wire, such as watch-makers use on watch cases. The hinge should be placed upon the upper surface of the plates so as to leave a shoulder to prevent the velum from falling to low. A delicate gold spring must be attached to the upper surface of the obturator and allowed to rest upon the velum so that the moment the muscles relax their hold upon the velum, it will be pushed down.

Thus much as to how an obturator or an artificial velum should be constructed for a typical case of congenital cleft palate. Various as may be the conditions, the same general principles underlie them all. There is but one improvement which remains to be made, and that is to produce an artificial velum which shall be the perfect shape of the natural one when at rest, and be made to assume when drawn up, the shape of a natural velum elevated. Who ever shall produce such an artificial velum, will indeed be entitled to the crown of perfection.—*Missouri Dental Journal.*

